# Comprehensive assessment of holding urine as a behavioral risk factor for UTI in women and reasons for delayed voiding

**DOI:** 10.1186/s12879-022-07501-4

**Published:** 2022-06-06

**Authors:** S. Jagtap, S. Harikumar, V. Vinayagamoorthy, S. Mukhopadhyay, A. Dongre

**Affiliations:** 1 Department of Microbiology, Pondicherry University, Puducherry, India; 2 Department of Community and Family Medicine, All India Institute of Medical Sciences, Deoghar, Jharkhand India; 3 Department of Community Medicine and Extension Programme, Pramukhswami Medical College, Karamsad, Gujarat India

**Keywords:** Questionnaire, Urinary tract infection, MDR, Uropathogen, Risk factors

## Abstract

**Background:**

Women of reproductive age group have greater predilection to urinary tract infections (UTI). Various risk factors increase the prevalence in women. Emergence of multidrug resistant uropathogens make clinical management of UTI challenging. Here we assess holding of urine as risk factor of UTI in women and reasons for delayed voiding. We also investigate the relationship between frequency of UTIs and overall behavioural features, menstrual hygiene and attitude of women towards their own health issues.

**Methods:**

A questionnaire based cross-sectional study was performed with 816 hostel residents with written consent. Self-reported data was statistically analysed using SPSS software. Urinalysis and urine culture were done for 50 women by random sampling to obtain the information on leading causative agents of UTI in the study population and their antimicrobial resistance profile.

**Results:**

The prevalence of UTI among the participants without risk factors was found to be 27.5 (95% CI: 24.4–30.7). Attitude of women towards their own personal health issues and use of public toilets showed a correlation with prevalence of infection. Delay in urination on habitual basis was found to be associated with UTI. Uropathogens isolated by random sampling were resistant to multiple drugs that are generally used to treat UTI.

**Conclusions:**

Holding urine for long time had proven to be an important risk factor and amongst different reasons of holding urine, holding due to poor sanitary condition of public toilets was the most common. Higher frequency of self-reported UTIs is related to holding of urine, behavioural features and attitude of women.

## Introduction

The bacterial colonizers at the urethral opening are flushed out by micturition. Urinary tract infection (UTI) is an inflammation of the urothelium as a result of pathogenic invasion, affecting lower and upper urinary tract. [1]. Women have a greater predilection for UTI. In the age group of 16–35, women are 35 times more likely to be affected by UTI than men [1]. Higher susceptibility in women is a function of basic anatomic factors (shorter urethra and its proximity to bacterial reservoirs in the rectum and vagina) as well as behavioral factors [1]. Behavioral factors help microbial uropathogens capitalize on female anatomic vulnerability [2]. Urinary tract infections (UTIs) in otherwise healthy individuals with no structural or neurological abnormalities are termed as uncomplicated UTIs that are differentiated into cystitis (lower UTIs) and pyelonephritis (upper UTIs) [3]. Typical symptoms of a lower urinary tract infection include urgency, frequency and dysuria. Several risk factors have been reported to be associated with lower UTIs (particularly women) including socio-demographic factors [4], age, history of UTI, and diabetes [5] caffeinated beverage consumption and alcohol [6], inadequate water intake [7], sexual activity [8], international travel [9], lifestyle factors and co-morbidities [10], spinal cord injury and catheterization [11]. The term UTI in this study is used to represent lower UTI (Cystitis).

Apart from the above mentioned factors, behavioral risk factors are also associated with UTI in women. Sedentary lifestyle > 6 h/day, delayed voiding and sanitary hygiene were associated with an increased risk of recurrent UTI in postmenopausal women [12]. We have explored to assess the other associated potential risk factors for UTI in women like behavioral features and attitude. We hypothesized that if a woman delays urination by holding urine on regular basis for various reasons, it increases her chances of suffering from UTI multifold. We undertook a research study to test this scientifically with statistical analysis and also to find out the different reasons behind holding urine. We conducted epidemiological survey among institutionalized women students followed by microbiological analysis of uropathogens. Although with low morbidity lower UTIs resolve quickly, there are certain issues related to it and hence they must not be ignored. One is economic burden and the other is the propensity to recur. Apart from this, the bacterial pathogen may ascend and cause upper urogenital tract infections which are serious and can be fatal [13].

The most dominant causative agent for both uncomplicated and complicated UTIs is uropathogenic *Escherichia coli* (UPEC). In uncomplicated UTIs, for the causative agents involved UPEC is followed in prevalence by *Klebsiella pneumoniae, Staphylococcus saprophyticus, Enterococcus faecalis*, group B *Streptococcus* (GBS), *Proteus mirabilis, Pseudomonas aeruginosa, Staphylococcus aureus* and *Candida* spp. [3].

One of the objectives of the current study was to assess the prevalent organisms in our campus and their drug susceptibility pattern as UTI pathogen spectrum and their drug resistance pattern vary according to geographical location.

## Materials and methods

### Study design

This was a cross sectional study conducted in eight ladies’ hostels of Pondicherry University during January 2019–April 2019. 816 women in reproductive age group participated in the study. The study was conducted to assess the potential risk factors for UTI in the selected women population which have experienced the institutionalized exposure for at least six months in the campus.

### Development of questionnaire

The questionnaire was designed to get unambiguous data from the participants. It was developed after comprehensive literature review and finalized by consulting epidemiologists and urologists, five each. It was first administered in a draft form to a sample of 25 women (which is 6% of estimated sample size) to verify practicality of using it for the survey [14].

The questionnaire comprised of 6 different domains: (a) Demographic profile to obtain information about educational qualification, religion, marital status and age, (b) Urinary symptoms with 15 questions of key UTI symptoms [10, 15, 16] (c) known risk factors for exclusion criteria (elaborated in data collection) as we wanted to focus on other new potential risk factors, (d) attitude of women towards their health, (e) personal behavioral features with questions about daily intimate hygiene and holding of urine and possible reasons for holding, [1, 12] and (f) menstrual hygiene [15]. The risk factors we assessed were personal intimate hygiene, holding urine and reasons for delaying voiding, and menstrual hygiene. These factors were not used for exclusion. Attitude of women towards their health was assessed by asking questions about their willingness to seek medical treatment for UTI and use of public toilets.

### Data collection and analysis

This was a cross sectional study conducted in eight ladies’ hostels of Pondicherry University during January 2019–April 2019. Prior written informed consent of all the participants was obtained. The study was carried out on approval by Institutional Human ethics committee. The data was collected from the participants in form of self- reported responses to the questionnaires. Data were collected and analyzed using SPSS for Windows 24.0 (SPSS Inc, Chicago, IL, USA). Data were presented as number and percent (%) for categoric variables. The data with p value < 0.001 only was considered to be statistically significant. The p-value was calculated for statistical analysis of each parameter studied. The inclusion and exclusion criteria were applied.

Women residing in the hostels in the university campus at the time of survey were randomly selected. Inclusion criteria: (a) residence in the campus for at least 6 months (b) willingness to participate in the survey with informed consent, (c) respondents that provided complete information on the questionnaire. After receiving responses from the participants a woman with any one of the following conditions was excluded from the analysis so that clear data can be obtained on the risk factors in question and can be analyzed to find their association with UTI if any. Exclusion criteria: Women who were already diagnosed with (a) diabetes, (b) chronic kidney disorder, (c) chronic neurological disorder, (d) recent hospitalization or antibiotic treatment (e) those who did not respond to questions about the known risk factors mentioned (a) to (d) and, (f) respondents with incomplete questionnaire data.

Estimation of sample size: The minimum number of participants for the survey were calculated using the formula.

$${\text{N}} = \frac{4PQ}{{d^{2} }},$$

N = Desired sample size for the study. P** = **Prevalence of UTI symptoms among women from the region from previous study: 20.4% [17].

$${\text{Q}} = (100 - {\text{P}})$$

d = Relative Precision (20% of P)

$$N=(4 \times 20.4 \times 79.6)/(4.08)2=390.$$

The estimated sample size was calculated to be 390. Thus a sample size of 410 women (390 + considering 5% non-response rate) was taken to increase the representativeness of the sample, minimize sampling errors, increase generalizability of the result and cater for any drop out. It was possible for us to get the more than the desired sample size because of abundance of subjects who meet the desired criteria.

The questionnaire comprised of objective questions to obtain information about signs and symptoms of UTI which include: urgency, hematuria, dysuria, incomplete urination, suprapubic pain, dribbling, urge incontinence, nocturia, urethral pain and straining. Reponses for any 3 mentioned sign and symptoms were considered to be positive for UTI.

### Urine-analysis

#### Pre culture examination of urine

Of all the participants in survey, after applying exclusion criteria 214 who were found to be UTI positive based on their responses in the questionnaire after, 50 were randomly recruited for urine sample analysis. They were asked to provide a specimen of clean catch midstream urine that was processed in the laboratory within two hours of collection. Urine samples were subjected to different laboratory tests and culture media for identification of the etiological agents. Pre culture examination of samples was carried out by performing tests: pH, color, turbidity; microscopic examination for presence of leukocytes, epithelial cells, blood cells using 40X objective.

### Urine culture

The samples with pyuria (WBC/leukocyte count) were cultured on sterile cysteine lactose electrolyte deficient (CLED) media. In this study any organism isolated with colony counts of greater than 10,000 cfu/ml of urine was considered significant and indicative of a UTI. Gram staining and motility tests were performed of the isolates obtained.

The biochemical tests were conducted according to the methods described by Bergey’s manual of determinative bacteriology [18]. Biochemical characterization of isolates was done by Indole test, catalase test, urease test, Citrate utilization test, TSI (Triple Sugar Iron) test, Phenylalanine Deaminase test, Novobiocin sensitivity test.

### Antibiotic sensitivity test

The Antimicrobial susceptibility test of the isolates was carried out using sterile Mueller–Hinton agar using disc diffusion (Kirby Bauer’s) method and interpretation was done after 15–20 h of incubation at 37 ℃ according to Clinical and Laboratory Standards Institute (CLSI) guidelines (https://clsi.org/standards/). Antibiotic disc of 20 antibiotics which were commonly used for the treatment of UTI were selected. Sensitivity testing was done for Amikacin (AK), Cefazolin (CZ), Cephalothin (CEP) Cefepime (CPM), Doxycycline Hydrochloride (DO), Tetracycline (TE) 30 µg each; Cefotaxime (CTX), Ciprofloxacin (CIP), Gatifloxacin (GAT), Moxifloxacin (MO) 5 µg each; Gentamicin (GEN), Norfloxacin (NX), Ampicillin (AMP), Streptomycin (S) 10 µg each; Nitrofurantoin (NIT), Carbenicillin (CB) 100 µg each; Co-Trimoxazole (COT 25 µg), Cefoperazone (CPZ 75 µg), Ampicillin/Sulbactam (A/S 10/10 µg), Piperacillin/Tazobactum (PIT 100/10 µg): all 20 discs on a single inert flat circular ring; Icosa UTI-1 from Himedia was used for the test.

## Results

### Prevalence of UTI

**Fig. 1:**
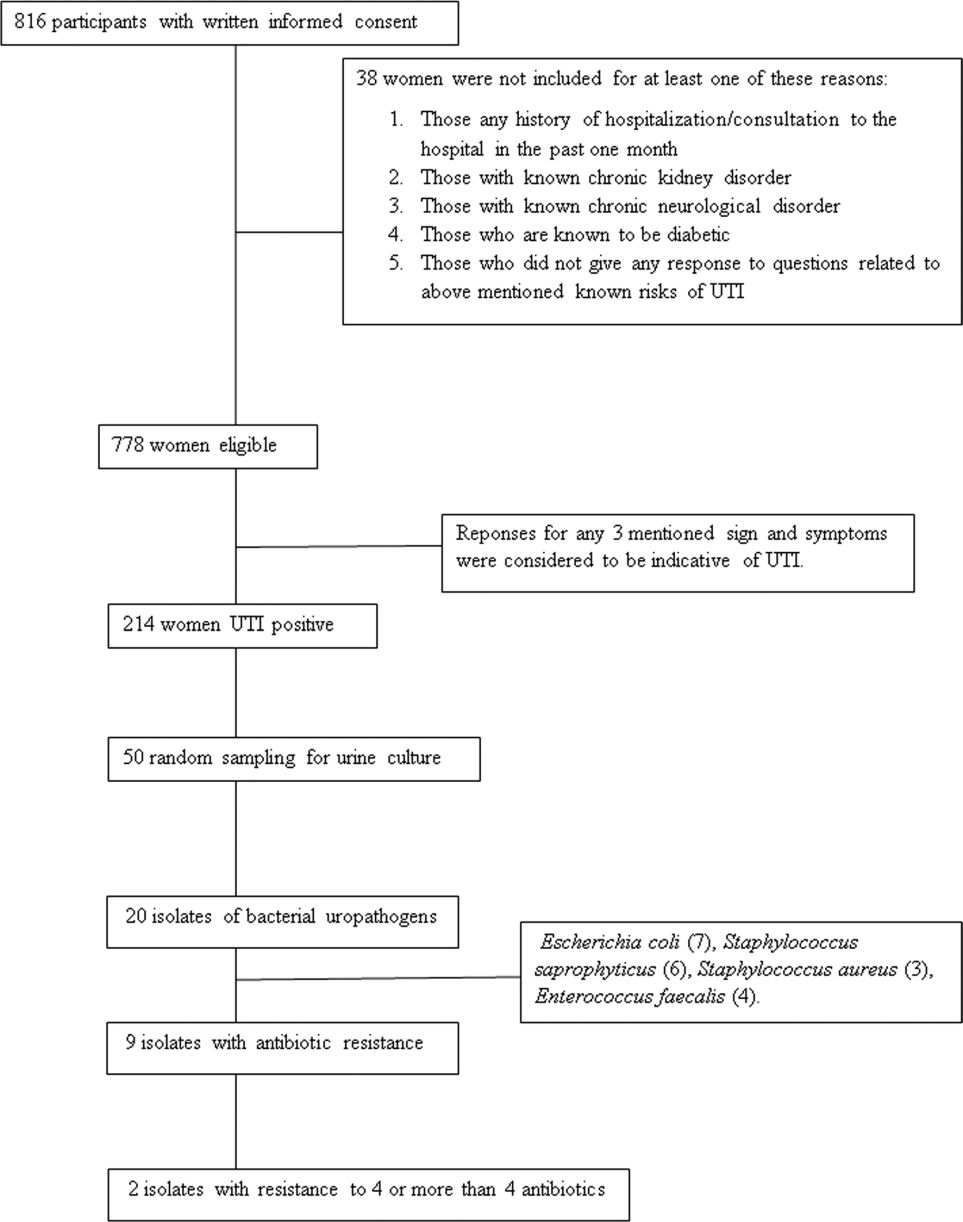
Work flow chart

On applying exclusion criteria for known risk factors and co morbid conditions for UTI like diabetes, kidney disorder, recent history of UTI/hospitalization or antibiotic treatment, out of 816 women, 778 qualified for further analysis to test our hypothesis. The scheme of the entire reserch work is outlined in the Fig. 1. The p value was calculated for each paramaeter studied and is mentioned in the respective tables. The p value > 0.001 was conidered to be non-significant.

**Fig. 2:**
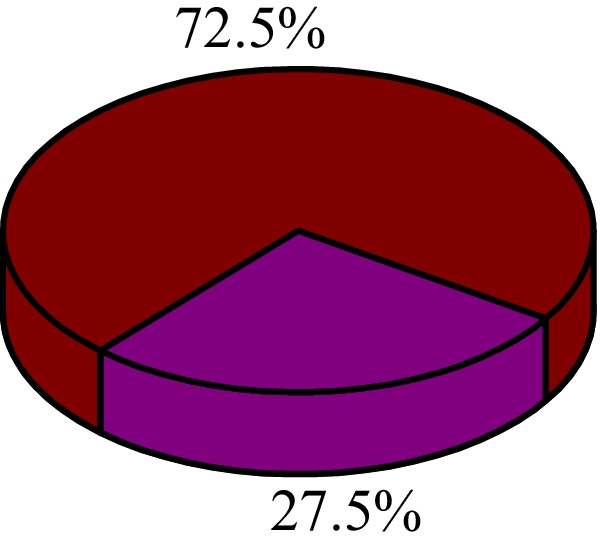
Prevalence of UTI in the study population

Using this criteria, 214 of 778 women were found to be UTI positive after applying exclusion criteria. The prevalence of UTI among hostel residing women who participated in this study was 27.5% with 95% Confidence Interval of 24.4–30.7 (Fig. 2).

The sociodemographic data of study population is given in Table 1. None of the sociodemographic characteristics (age, education, marital status, and religion) found to have statistically significant correlation with UTI.

**Table 1:** Association of socio-demographic factors with UTI status among the study subjects (N = 778)

Demographic characteristics	N	UTI			
		n	%	Unadjusted OR (95% CI)	P value
Age in years					
17–20 (teenage)	206	58	28.2	1 (Reference)	NA
21–23 (college going)	383	99	25.8	0.9 (0.6–1.3)	0.55
24–32 (adult)	189	57	30.2	1.1 (0.7–1.7)	0.66
Educational qualification					
Undergraduate	42	13	25	1 (Reference)	NA
Postgraduate	559	150	26.8	1.1 (0.6–2.1)	0.77
Ph.D	167	51	30.5	1.3 (0.6–2.7)	0.45
Marital status					
Unmarried	761	209	27.5	1 (Reference)	NA
Married	17	5	29.4	1.1 (038–3.2)	0.86
Religion					
Hindu	587	165	28.1	1 (0.36–2.9)	0.97
Christian	116	29	25	0.9 (0.3–2.6)	0.80
Muslim	57	15	26.3	0.9 (03–3.1)	0.90
Others	18	5	27.8	1 (Reference)	NA

The most common presenting symptoms in the UTI positive group were frequency (64%), flank pain (48%), urgency (47%), dribbling (38%), and incomplete urination (35%). Less frequent but still substantial symptoms were dysuria (28%), hesitancy (24%), urge incontinence (26%), pain in urethra (18%) and straining (18%). Less commonly reported symptoms were hematuria (3%) and fever (8%) (Table 2).

**Table 2:** Frequency table of urinary symptoms in UTI positive participants (N = 214)

Urinary symptom in last one month		Number	%
a	A sudden desire to urinate, which is difficult to hold (urgency)	100	47
b	Urinary leakage, because you cannot hold the sudden desire to urinate (urge incontinence)	56	26
c	Delay in starting urinary stream (hesitancy)	51	24
d	Need to strain to keep urinary stream(straining)	38	18
e	Feeling of incomplete emptying bladder after you urinate	74	35
f	Leakage of urine after you finish urinating (dribbling)	81	38
g	Pain in the bladder or lower abdomen or supra pubic region	102	48
h	Pain in the urethra	38	18
i	Burning on urination (dysuria)	61	28
j	Pain on urination (dysuria)	36	17
k	Blood in urine (hematuria)	7	3
l	Fever associated with any of the above symptom	17	8
m	Any of the above symptom appears after menstruation	82	38
n	Frequency of urination	137	64

Binary analysis of our results showed that known risk factors like sexual activity, caffeinated drinks, intimate and menstrual hygiene had no correaltion with UTI (Table 3). The number of sexually active women was very low in the study group.

**Table 3:** Association of known risk factors with UTI status among the study subjects (N = 778)

Risk factor	N	UTI			
		n	%	Unadjusted OR (95% CI)	P value
Consumption of caffeinated drinks					
Yes	603	165	27.4	1.1 (07–1.5)	0.87
No	175	49	28	1 (Reference)	NA
Sexual activity					
Not Active	753	208	27.6	1.2 (0.5–3)	0.69
Active	25	6	24	1 Reference)	NA
Intimate hygiene (N = 756)					
Appropriate	417	123	29.5	1.3 (0.9–1.7)	0.15
Inappropriate	339	84	24.8	1 Reference)	NA
Menstrual hygiene (N = 755)					
Appropriate	648	173	26.7	1 Reference)	NA
Inappropriate	107	35	32.7	1.3 (0.9–2.1)	0.20

### Personal hygiene and UTI

The data shows amongst UTI positive women, 27% of women with UTI do not wash the vagina after each urination. Though intimate hygiene showed no statistically significant correlation with UTI, 63.6% of women with UTI self reported poor perineal hygiene; washing perineal area from back to front (Table 4).

**Table4:** Frequency table of intimate hygiene in UTI positive participants (N = 214)

Hygiene parameter	UTI	
	n	%
Washing vagina daily	209	97.7
Washing intimate area with water after urination each time	155	72.4
Wiping from back to front	136	63.6
Using tight fitting undergarments	57	26.6

Around 20–30% of women suffer recurrent UTI (rUTI) within 6 months of a first episode and some have an average two or three additional UTIs within a year. The source of etiological agent of subsequent UTI is often the gastrointestinal tract which is the result of poor perineal hygiene [19]. Stapleton described the primary route of UTI reinfection in adult women as originating in the intestine [1]. Therefore knowing high risk factors which make women most likely to suffer from rUTI is important.

### Menstrual hygiene and UTI

Though menstrual hygiene showed no statistically significant correlation with UTI, 38% of women with UTI self reported to have UTI signs and symptoms after mensturation. Though 87.4% women use sanitary pads during menstruation, 76% of women change pads only 2–3 times a day. Use of soap and water to clean genital area was found to show strong correlation with UTI. Of UTI positive group as high as 65% women use soap and water to clean genital area (Table 5).

**Table 5:** Frequency table of menstrual hygiene in UTI positive participants (N = 214)

Hygiene parameter	UTI	
	n	%
(a) Use of sanitary pads for menstrual protection	187	87.4
(b) Frequency of changing pad (More than 4/day)	162	75.7
(c) Use of Soap and water/vaginal wash to clean genital area	139	65

### Holding of urine and UTI

It is observed that women hold back urine from not voiding which has prooved to be a potential risk factor for UTI in this study (Table 6).

**Table 6:** Multivariate binary logistic regression showing association between UTI and other independent variables (N = 778)

Risk factor	N	UTI			
		n	%	Unadjusted OR (95% CI)	P value
History of holding urine in various situation					
Yes	657	192	29.2	2.0 (1.2–3.3)	< 0.001*
No	121	22	18.2	1 (Reference)	NA
Past history of UTI					
Yes	62	33	53.2	1 (Reference)	NA
No	716	181	25.3	3.7 (2.1–6.4)	< 0.001*

We also tried to understand the reasons for holding back urine by asking different questions in the questionnaire; the results are presented in Table 7. Less frequent but still substantial risk factors for UTI were sheer laziness (42%), hectic work schedule (43%) and hesitancy to excuse oneself in a mixed crowd (42%). Women reported to delay urination during long, non stop travels (89%) as well as due to unavailability of public toilets (88%) when outside home for a long time. Apart from this it is important to note that 91% women that were found to be suffering from UTI hold back urine because they think that the public toilets are not clean enough; the sanitory conditions of public toilets not agreeable to them. So along with the infrastructural inadequacies of public toilets, the chintzy attitude of women was found be strongly associated with the UTI.

**Table 7:** Frequency table of reasons for holding urine in UTI positive participants (N = 214)

Reasons for holding urine	UTI	
	n	%
During long, non-stop travels	190	88.8
Unavailability of toilets	187	87.4
Hesitation to excuse oneself to reach washroom; particularly in mixed gathering	91	42.5
Due to hectic work schedule	93	43.5
Sheer laziness	91	42.5
Sanitary conditions of public toilets are not agreeable	194	90.7

### Womens’ attitude towards health and UTI

**Fig. 3:**
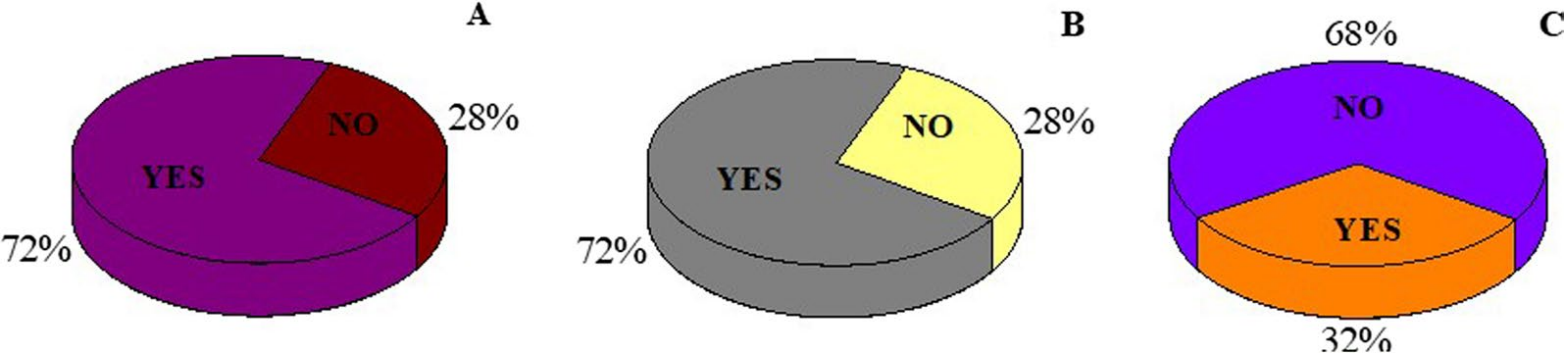
Attitude of women towards own health. **A** Do you think symptoms are serious enough to consult a doctor?. **B** Would you consult a doctor if you have symptoms?. **C** Have you consulted a doctor in the past when you had symptoms?

From this study we found that among the prevalence group, though majority (72%) of women think that signs and symptoms are serious enough to consult a doctor and many (72%) responded that they would consult a doctor if they have it but interestingly only 31.8% women actually consulted a doctor after having the symptoms of UTI in the past (Fig. 3). This explains why we could get isolates of MDR uropathogens from the participants.

### Urine examination and analysis

Of 50 samples analyzed 31 showed indication of UTI by microbiological analysis based on different parameters like WBCs count and cfu/ml. Twenty isolates were considered to be undoubtedly uropathogens which were identified by various biochemical tests recommended and were further tested for antibiotic resistance.

From 20 isolates obtained from urine samples collected most commonly isolated bacteria were *Escherichia coli* (35%), *Staphylococcus saprophyticus* (30%), *Staphylococcus aureus* (20%), *Enterococcus faecalis* (15%). Generally it is found that *E.coli* is the leading cause of UTI [16]. In our study it is found that among the samples tested, the number of isolates of *E.coli* were followed by *Staphylococcus saprophyticus*.

#### Antibiotics resistance of uropathogens

Amongst 20 isolates 9 were antibiotic resistant (3 were of *Staphylococcus aureus*, 4 of *Staphylococcus saprophyticus* and 2 of *E. coli*). ESBL and fluroquinolone resistance was common. The two isolates of *Staphylococcus aureus* were found to be multidrug resistant; resistant to four and more than four antibiotics tested: one is resistant to AMP, COT, DO, GAT, NIT, NX and TE whereas other is resistant to AMP, CZ, CEP and NIT.

## Discussion

Our study provides insight about the symptomatic detection of UTI in young women from self-reported symptoms and its correlation with proposed risk factors like personal behavioural features and attitude about their personal health and as well as their menstrual hygiene. Various aspects of epidemiology of UTI in college women is studied by Foxman et al. like menstrual protection and intimate hygiene [20–22]. UTI among college students is studied by different groups for prevalence, drug resistance and associated risk factors. [7, 17, 23].

Although we believe that holding back of urine for long time increases the chances of UTI by increasing the growth of microbes in bladder, but there is no scientific and statistical evidence in published literature till date. We hypothesized that holding urine is one of the major risk factor of UTI. We were also curious to know the possible reasons for holding urine.

In this study first time statistical information about attitude of women towards personal health and holding urine (delaying urination) on regular basis was obtained. The most interesting finding of the current research work is that there is a significant relationship between holding urine and prevalence of UTI. Women admitted to hold urine “always” or “sometimes” due to various reasons. Some of them are beyond their control and under those circumstances they are helpless like: during long non-stop travels with no washroom facilities are not available; 88.8% of women report to hold urine under such conditions. Similarly, unavailability of ladies’ toilets in public places was also found to be reason for 87.4% of women to hold urine. Apart from these infrastructural inadequacies, women reported to hold urine for other reasons too which are indicative of their attitude. 90.7% of women prefer to delay urination even if public toilets are available because they think that the sanitary conditions of public toilets are not agreeable to them. Cleanliness is a subjective term and apparently majority of women have fussy attitude towards use of public toilets. Holding urine once may not predispose a woman to UTI as harboured pathogens near urethral opening are flushed out in the process of micturition. If a woman makes it habit to hold urine for one or the other reason, it would give enough time for a bacterial pathogen to ascend and cause infection of kidneys which can be a serious health concern or even fatal. Periurethral colonization with the causative bacterial strain increases incidence rate of UTI [1]. rUTI can occur either by ascending reinfection (the source is outside genitourinary tract) or by reinoculation (the source is within genitourinary tract). Based on the outcome of our studies, we would recommend that women should not hold urine from voiding; by doing so and implementing other corrective behavioural measures they can minimize the risk of UTI and rUTI.

There are reports that comment that avoidance of urine holding isn’t a demonstrable beneficial risk reduction strategy [1]. Delayed voiding has been reported to be a behavioural risk factor of recurrent urinary tract infection in Chinese postmenopausal women [24]. Our research clearly indicates that urine holding on regular basis is associated risk factor for UTI in women. We also investigated in detail the reasons for holding urine some of which are related to infrastructural issues like that of unavailability of toilets but some attribute to the fastidious outlook of women.

On urine analysis, amongst 20 isolates 9 were antibiotic resistant. Drug resistant UTI are responsible for longer stay in hospitals, increased expenses, higher rates of treatment failures, and mortality [25]. Independent research studies by Hsueh et al. [26] and Tandogdu and Wagenlehner [5] emphasized on epidemiological data on local resistance patterns as according to geographical location UTI pathogen spectrum and their resistance to common antibiotics vary [5, 26]. In Peru, ESBL (extended-spectrum b-lactamase) *E. coli* accounted for more than 40% of CA (community acquired)—UTIs during 2015 [27]. In Hungary treatment of drug resistant uropathogens *Citrobacter, Enterobacter*, and *Serratia* species (CES bacteria) is challenging for clinicians [28].

Data from 13 countries in the Asia–Pacific region shows that antibiotic resistance is a serious problem [29]. In the Asia–Pacific region, around 33% of urinary *E. coli* isolates exhibited ESBL-resistance with highest prevalence in India (60%), High level of drug resistance present a therapeutic challenge. For selection of empiric therapy for UTI, accurate population surveillance data with updates is needed [11]. These results underline the importance of antimicrobial stewardship [25]. Urinary isolates of *E. coli* with ESBL resistance is reported from France [22, 30], Thailand [31], India [32, 33] and other countries. A retrospective analysis of around 50,000 hospitalised patients with UTI indicates the presence of organisms resistant carbapenem which are used as “antibiotics of last resort” to treat drug resistant bacteria [34]. This not only has worse outcomes but also alarms the worrisome situation for UTI therapy. The resistance to the commonly prescribed drugs warrants antibiotic stewardship and implementation of GLASS (Global Antimicrobial Resistance Surveillance System) launched by World Health Organization (WHO). To limit emergence of drug resistant microorganisms and to maximize clinical cure antimicrobial stewardship is required [25]. Implementation of GLASS in Thailand in 2015 has been found to be more beneficial than laboratory-based surveillance [32]. At the same time, alternative treatments that are rationally designed are need of the hour. Perturbations/dysbiosis in the urogenital microbiome [35] are associated with UTI implying that correcting dysbiosis could possibly be an effective preventive measure for UTIs and rUTIs.

One more thing to be noticed in this context is attitude of women towards their own health. Though many of the participants responded with answer that the UTI symptoms are serious enough to consult a doctor (72%) but interestingly very few (32%) of them actually have seen a doctor in the past in such case (Fig. 3) that indicates why we could get isolate of MDR (multiple drug resistant) uropathogens from the participants.

Our data suggests that women are negligible about their personal health. Qualitative interview study conducted by Leydon et al. in UK on women’s views on UTI found that women prefer to wait and self-manage UTI since onset of symptoms before visiting a doctor [36]. It was also found that women avoid taking antibiotics and they attribute their UTI to poor cleanliness and negligence.

In younger women recurrence of UTIs within 6 months is common. Recurrent UTIs (rUTIs) present societal as well as personal burden. The psychological effects have a negative impact on quality of life (QoL) and self-esteem in young women. Recurrent UTIs are associated with symptoms of anxiety and depression [37]. To minimize the incidence of UTIs and rUTIs, it is important to have data on prevalence of the infection and associated risk factors in a given population. This can help to devise intervention and/or awareness programs if suitable. In a study conducted at Boston, USA, depressive symptoms were associated with increased odds of lower UTI across all sex and racial/ethnic groups [10]. Girls are more prone to develop renal scars as a result of recurrent UTIs in comparison with boys [35]. Therefore, it is important to identify young women at high risk for recurrent UTIs and develop interventions to minimize the risk of rUTIs. A (Theory of planned behavior) TPB-based educational awareness talks on promoting the preventive behaviors of UTI was found to be effective in minimizing UTI in Iran [38], we recommend using such strategy as a trial in institutionalized stays. The study limitation is that it was done using a single site (university campus) with a limited age group range and it cannot be used to generalize the results of the study for the entire women population. As information about signs and symptoms of UTI was provided by the participants was entirely self-reported, possibility of bias cannot be ruled out. Additionally, larger number of samples should be analyzed to create data on uropathogens spectrum and understand their antimicrobial resistance pattern.

## Conclusions

The prevalence of UTI among the participants was found to be 24.3%. In conclusion, there are substantial burdens of UTI in the hostel-dwelling women population, prompting growing interest in the issues of UTI. The results showed that holding back urine and persnickety attitude towards use of public facilities are significantly associated with UTI. Effective preventive measures to reduce the prevalence of MDR-UTI are needed. The study limitation is larger number of samples should be analyzed to create data on uropathogens spectrum and their antimicrobial resistance.

## Data Availability

The datasets used and/or analyzed during the current study are available from the corresponding author on reasonable request.

## Abbreviations

UTI: Urinary tract infections
WBC: White blood cell
CLED: Cysteine lactose electrolyte deficient
cfu/ml: Colony forming units per mili litre
TSI: Tri sugar iron
rUTI: Recurrent UTI
ESBL: Extended-spectrum b-lactamase
GLASS: Global Antimicrobial Resistance Surveillance System
WHO: World Health Organization
MDR: Multiple drug resistance
TPB: Theory of planned behaviour
QoL: Quality of life
